# Prehospital assessment of perinatal patients by ambulance clinicians: development, implementation, review and national application

**DOI:** 10.1186/s12245-026-01158-5

**Published:** 2026-04-16

**Authors:** Camella Main, Joanna Shaw, Lydia Burgess, Stephanie Heys, Susan Rhind, Tony Kelly

**Affiliations:** 1 London Ambulance Service NHS Trust, 220 Waterloo Road, London, SE1 8SD UK; 2 East Midlands Ambulance Service NHS Trust, London, UK; 3 North West Ambulance Service NHS Trust, Lancashire, UK; 4 National Health Service England, London, UK

## Abstract

**Background:**

Prehospital maternity care is complex due to limited diagnostic equipment and access to pertinent information, constraints on resources, environmental challenges, minimal education and exposure to maternity cases. NEWS2 is used to assess adults in the prehospital setting but is not validated to assess perinatal patients. This paper describes the development, implementation, review, refinement and national implementation of a bespoke prehospital assessment tool which accounts for the altered physiology of perinatal patients.

**Methods:**

An initial modified Delphi approach, using expert focus groups, reached a consensus on the early warning parameters and other red flags required for a prehospital maternity decision tool. The tool was implemented and retrospectively applied to anonymised patient data in a large urban ambulance service in the UK. A further modified Delphi approach was then used to revise the tool to ensure national applicability.

**Results:**

The initial modified Delphi resulted in a tool which included maternity early warning score parameters and other red flags. Following implementation within one ambulance service, data from 251 ambulance clinical records of pregnant or recently pregnant women identified a high proportion of women with abnormal signs and symptoms which would not have been identified using NEWS2. Through a second modified Delphi, a national tool underpinned by national MEWS parameters alongside additional time-critical red flags was produced.

**Conclusion:**

A national prehospital maternity decision tool was created using modified Delphi methodology. Further research is required to formally validate the tool and to understand the effectiveness when screening for perinatal deterioration in the prehospital setting.

**Supplementary Information:**

The online version contains supplementary material available at 10.1186/s12245-026-01158-5.

## Background

Despite increased focus on maternal safety, subtle but critical signs of clinical deterioration in perinatal women continue to go unrecognised contributing to persistent and preventable rates of maternal morbidity and mortality in high-income countries [1, 2]. In the United Kingdom (UK), where approximately 1% of 999 calls are for pregnancy or childbirth related reasons [3], this burden is unevenly distributed: Black and Asian women and those from deprived areas are disproportionately affected [2]. Perinatal patients requiring help from the ambulance service are often at risk of health inequalities and require a bespoke assessment by prehospital teams.

The Royal College of Physicians produced NEWS2 to ensure a consistent approach to assessment of patients and early recognition of deterioration [4, 5]. Whilst this early warning score has been validated in prehospital and hospital settings, it should not be used in perinatal patients due to their altered physiology [4]. Physiological changes in pregnancy, such as increased heart rate, expanded plasma volume, and altered respiratory physiology mean that deterioration can present subtly giving a normal NEWS2 score [6, 7].

A national maternity early warning score (MEWS) chart has also been developed to aid identification of deteriorating pregnant patients in maternity units [7]. The chart is designed to help perinatal clinicians to detect trends, escalate and plan treatment. However, this has not been validated for use in the prehospital setting.

When ambulance services respond to perinatal women, patients may not have previously accessed antenatal care because of language challenges, discrimination, cultural differences, and/or lack of continuity of care which has consequential impacts on poor outcome and health inequalities [8, 9]. In addition, the ambulance service does not have access to clinical records to optimise history taking. Therefore, prehospital services require clear and specific guidance for clinicians to effectively assess and manage pregnant and postnatal women based on the information available to them in the prehospital setting, and ambulance clinicians need to have a low threshold for timely escalation to definitive care.

Prehospital maternity care is an underexplored area of healthcare and often absent in strategic programmes to improve maternity care. This study aims to describe the process undertaken to develop, implement, review and apply a prehospital maternity decision tool on a national scale.

## Methods

### Design

This paper details a 3-stage approach to the advancement of bespoke prehospital guidance for the assessment of perinatal women who call an ambulance in the UK, specifically: development of the tool, implementation and review, and refinement and national application.

### Development of the tool: Modified Delphi 1

In the absence of rigorous scientific research and given the urgent need for guidance [10], a 3-step modified Delphi method was used to establish consensus guidance for out of hospital assessment of perinatal patients. 22 experts representing ambulance service midwives, paramedics, non-registrants of different grades and patient safety teams were invited to participate on the expert panel. The panel was recruited based on their expertise in prehospital and maternity care, using the snowball recruitment method. The first round of the modified Delphi, conducted in June 2022, was replaced by an online focus group, to facilitate the unstructured gathering of opinions and rich qualitative discussion; it was vital that individuals could bring their experiences and understanding of prehospital perinatal care. This meant anonymity of participants was not upheld. Following the initial focus group, statements were compiled and 2 further rounds of focus groups were held until an expert consensus on the elements to be included in the guidance was reached.

The setting was a large English urban ambulance service with over 5,000 operational clinicians attending more than a million patients face-to-face annually. The focus groups included discussion of approximately 100 incidents from the previous 3 years which had been investigated using Systems Engineering Initiative for Patient Safety (SEIPS) methodology [11] where the ambulance service had been called to perinatal women and the outcomes were suboptimal or a “near miss” was identified.

### Implementation and review

The agreed elements were converted to a tool which was available to all ambulance clinicians in the 999-control room and those attending face-to-face emergencies. Paper copies were accessible within the birth packs on ambulances, and an electronic version was made available on the clinical guidelines portal. Clinicians were recommended to use the tool when attending pregnant, suspected pregnant or recently pregnant patients (within 4 weeks) to make an initial assessment and determine whether emergency conveyance to the maternity unit was required. Introduction of the tool was widely communicated via clinical notifications and webinars.

Previously collected anonymised clinical data, gathered in line with normal care, for 251 patients who had called the ambulance service during the first 6 days of March 2024, were collated and stored following Good Clinical Practice Guidelines [12]. Data were analysed using descriptive statistics in IBM SPSS Statistics for Windows, Version 27 (Armonk, New York, United States of America) to determine what clinical observations were recorded for perinatal patients. Where not already recorded, both the tool and NEWS2 were retrospectively applied to determine the severity of the patient’s condition and allow for comparison of the tools. If clinical data required to determine severity was missing, these cases were excluded from the analysis. Ethical approval and informed consent were not required due to the secondary use of the data. Strengthening the Reporting of Observational Studies in Epidemiology (STROBE) guidelines were used to guide the reporting of this paper [13].

### Refinement and national application: Modified Delphi 2

The findings from the review fed into the 8 focus groups that occurred during 2024. The focus groups consisted of wide range of stakeholders from across the UK. 48 individuals from National Health Service (NHS) England, air ambulance charities, ambulance services, maternal medicine networks, obstetric anaesthesia, voluntary organisations, acute maternity services, secondary care, general practice and patient safety experts were invited to review the tool during development. The snowball recruitment method was again used to recruit the panel of experts in prehospital and maternity care. This additional modified Delphi approach was used to iteratively develop the prehospital maternity decision tool to ensure national applicability.

Women and families were also invited to review and critique the tool which was incorporated into national clinical practice guidelines in October 2024.

Guidance on Conducting and REporting DElphi Studies (CREDES) was used to guide the reporting of the development and revisions of the tool [14].

## Results

### Development of the tool

The focus groups in Modified Delphi 1 highlighted that the national MEWS chart is not suitable for use in the prehospital setting for several reasons. There are several elements of the MEWS chart that are not transferable to the prehospital setting due to the requirement for diagnostics and fetal monitoring. For example, a MEWS of two due to tachypnoea in an inpatient setting may initiate further monitoring of trends over time because immediately life-threatening causes have been excluded. In the prehospital setting, clinicians cannot exclude all the pathological causes of tachypnoea in pregnancy (for example, pulmonary oedema, chorioamnionitis, or peripartum cardiomyopathy) so this must instead initiate a time critical conveyance.

Findings showed ambulance clinicians, who were more familiar applying NEWS2, were inexperienced in assessing pregnant women; they often defaulted to using NEWS2 in these cases, despite it not being recommended for pregnant patients. The patient safety investigations discussed in the focus groups highlighted that the use of NEWS2 led to clinicians having a falsely low level of concern regarding the clinical presentation of some pregnant or peripartum women, resulting in a reduced sense of urgency.

There were also several presentations where the clinicians were falsely reassured due to the patient compensating due to their altered physiology. For example, there were several cases of bleeding in the third trimester of pregnancy where extended time on scene may have contributed to a poor outcome. Clinicians knew that bleeding in the third trimester of pregnancy was abnormal, but in most cases, the woman’s observations were found to be within acceptable NEWS2 parameters leading to false reassurance. It was also noted that in non-obstetric presentations, such as traumatic injury, altered physiology meant that the patient’s physiological response to the insult was slower compared to non-pregnant patients, which initially led to reduced concern during triage.

The findings from the Modified Delphi 1 and final agreed elements included in the guidance is shown in Table 1.

**Table 1:** Findings from modified Delphi 1

**Classifications agreed**	**Clinical significance agreed**
Green flags	Normal
Amber flags	2 amber flags are equal to 1 red flag
Red flags	“Abnormal” Requires time critical conveyance
**Element for inclusion**	**Parameters agreed**
Respiratory rate (per minute)	Green flag: 10–21 Red flag: below 10 or above 21
Pulse rate (beats per minute)	Green flag: 60–99 Amber flag: 51–59 or 100–110 Red flag: below 51 or above 110
Oxygen saturations (%)	Green flag: 95–100 Red flag: below 95
Systolic blood pressure (mmHg)	Green flag: 101–135 Amber flags: 94–100 or 136–144 Red flags: below 94 or above 144
Diastolic blood pressure (mmHg)	Green flag: 62–88 Amber flags: 57–61 or 89–96 Red flags: below 57 or above 96
Temperature (degrees Celsius)	Green flag: 36.2–37.2 Amber flags: 35.7–36.1 or 37.3–37.4 Red flags: below 35.7 or above 37.4
Cardiac symptoms	Red flags: chest pain or shortness of breath
Bleeding less than 20 weeks	Red flag: maternity sanitary pad soaked within 30 min (50mls)
Pain less than 20 weeks	Red flags: shoulder tip pain or one sided abdominal pain
Bleeding more than 20 weeks	Red flag: any vaginal bleeding
Pain more than 20 weeks	Red flags: constant abdominal pain or contractions less than 37 weeks
Neurological more than 20 weeks	Red flags: Any seizures after 20 weeks gestation

### Implementation and review

The elements agreed for inclusion in the guidance were compiled into a regional prehospital decision tool which was introduced as a clinical guideline on the electronic guidelines portal for one ambulance service in January 2023. Clinicians were informed about the tool through training, clinical notices and webinars.

Of the 251 perinatal women attended during the 6-day data collection period, mean patient age was 30 years, ranging from 16 to 45 years. Where stated, 66 patients were White (45.5%), 32 Asian (22.1%) and 32 Black (22.1%). 126 (50.2%) were 20 weeks pregnant or more, 59 (23.5%) were less than 20 weeks pregnant, and the remaining 45 (17.9%) were up to 4 weeks postpartum. For 21 women (8.4%), gestation was unknown.

Clinical observations recommended by the tool were well documented, specifically: respiratory rate (*n* = 250/251, 99.6%); oxygen saturation level (*n* = 244/247, 98.8%), pulse rate (*n* = 247/247, 100%); blood pressure (*n* = 244/246, 99.2%), and temperature (*n* = 225/246, 91.5%). The presence or absence of most red flags were also recorded for the majority of women, including: cardiac symptoms such as chest pain (*n* = 160/249, 64.3%) and shortness of breath (*n* = 178/250, 71.2%); vaginal bleeding (*n* = 220/250, 88.0%); pain (*n* = 230/248, 92.7%), and seizure activity (for women more than or equal to 20 weeks pregnant and for postpartum women; *n* = 31/169, 18.3%).

NEWS2 was calculated for 186 women; 14 (7.5%) had a NEWS2 *≥* 5 and 172 (92.5%) a NEWS2 < 5 (which in adult patients who are not perinatal would indicate possible non-conveyance). Of these 172 women, 148 (86.0%) were identified using the tool as having at least one red flag, or two or more amber flags. Therefore, their time-critical nature would not have been identified using NEWS2. 141 (95.3%) presented with red flags, the majority of whom (*n* = 108, 77.3%) had multiple red flags. The most common red flag identified was constant abdominal pain (*n* = 52, 36.9%) (for 12 of these women this was their only red flag symptom), followed by an abnormal heart rate (*n* = 33, 23.4%) and one-sided abdominal pain for women less than 20 weeks pregnant (*n* = 31, 22.0%). A further 7 women’s time critical nature was identified by a combination of 2 or more amber flags, with 5 of these relating to an abnormal blood pressure (Table 2).

**Table 2:** Presence of red and amber flags for perinatal women with NEWS2 < 5 (*n* = 148)

Red flags	Women with > 1 red flag* (*n* = 108)	Women with a singular red flag (*n* = 33)
Constant abdominal pain > = 20 weeks & postpartum	40 (27.0%)	12 (8.1%)
Heart rate, < 51 or > 110 bpm	29 (19.6%)	4 (2.7%)
One-sided abdominal pain < 20 weeks	27 (18.2%)	4 (2.7%)
Systolic blood pressure, < 94 or > 144 mmHg	30 (20.3%)	1 (0.68%)
Diastolic blood pressure, < 57 or > 96 mmHg	24 (16.2%)	-
Any vaginal bleeding > = 20 weeks	17 (11.5%)	5 (3.4%)
Temperature, < 35.7 or > 37.4 C	16 (10.8%)	1 (0.68%)
Respiratory rate, < 10 or > 21 /min	15 (10.1%)	-
Chest pain	12 (8.1%)	1 (0.68%)
Sanitary pad soaked (50mls) within 30 min < 20 weeks & postpartum	12 (8.1%)	1 (0.68%)
Contractions (< 37 weeks)	10 (6.8%)	-
Shortness of breath	5 (3.4%)	2 (1.4%)
Shoulder tip pain < 20 weeks	2 (1.4%)	-
Oxygen saturation, < 95%	1 (0.68%)	1 (0.68%)
Any seizures ≥ 20 weeks	1 (0.68%)	1 (0.68%)
**Amber flags**	**Women with no red flags and** **≥** 2 amber flags* (n = 7)	
Systolic blood pressure, 94–100 or 136–144 mmHg	5 (3.4%)	
Diastolic blood pressure, 57–61 or 89–96 mmHg	5 (3.4%)	
Temperature, 35.7–36.1 or 37.3–37.4 C	3 (2.0%)	
Heart rate, 51–59 or 100–110 bpm	2 (1.4%)	

*Women presented with multiple flags. Therefore, the sum of the number of flags exceeds the number of women

### Refinement and national application

During modified Delphi 2, expert panel members shared their experience of learning from patient safety investigations nationally and discussed the health inequalities that exist within the population of perinatal patients calling the ambulance service. Further consideration of what was possible within the UK prehospital setting such as exclusion of serious illness was also a crucial intention of the focus groups.

Expert consensus agreed that the national MEWS parameters [7] should be used in a flag system, alongside several other red flags identified as signs and symptoms that would require a time critical assessment in a maternity unit. The MEWS parameters are designed to be used from conception to 4 weeks post-pregnancy [7] so it was agreed that the tool would be recommended for perinatal patients during this timeframe.

The final tool was reviewed by the Joint Royal Colleges Ambulance Liaison Committee Guideline Development Group and is now available to all UK ambulance clinicians through the national clinical guidelines application [15, 16]. It was launched in all 13 UK ambulance services using webinars, education days and regional communication. Several events were held for system partners including maternity services and emergency departments to ensure that they were aware of the bespoke assessment that perinatal patients have in the prehospital setting prior to their admission. These events emphasised the importance of hospital services undertaking a further holistic triage of the woman following handover, with the diagnostics, expertise and fetal monitoring available. Table 3 shows the results of modified Delphi 2.

**Table 3:** Findings from modified Delphi 2

**Agreed classifications**	**Clinical significance**	**Development from modified Delphi 1**
Green flag	Low concern	Wording aligned to national MEWS.
Amber flag	Medium concern 2 amber flags are equal to 1 red flag	Wording aligned to national MEWS.
Red flag	High concern Requires time critical conveyance, minimise time on scene	Wording aligned to national MEWS. On scene prompt added.
**Element for inclusion in national tool**	**Parameters agreed**:	**Development from modified Delphi 1**
Respiratory rate (per minute)	Green flag: 9–21 Amber flags: 7–8 or 22–24 Red flags: below 7 or above 24	Parameters aligned to national MEWS. Medium concern parameters added.
Pulse rate during pregnancy and up to 48 h after birth (beats per minute)	Green flag: 71–112 Amber flags: 63–70 or 113–121 Red flags: below 63 or above 121	Parameters aligned to national MEWS. Pulse rate divided into pregnancy and up to 48 h after birth and from 48 h after birth.
Pulse rate from 48 h after birth (beats per minute)	Green flag: 58–98 Amber flags: 51–57 or 99–107 Red flags: below 51 or above 107	Parameters aligned to national MEWS. Pulse rate divided into pregnancy and up to 48 h after birth and from 48 h after birth.
Oxygen saturations (%)	Green flag: 95–100 Amber flag: 93–94 Red flags: below 93 or any oxygen requirement	Parameters aligned to national MEWS. Any oxygen requirement added including when administered for sepsis, postpartum haemorrhage etc.
Systolic blood pressure (mmHg)	Green flag: 101–135 Amber flags: 94–100 or 136–144 Red flags: below 94 or above 144	No changes – already aligned to national MEWS.
Diastolic blood pressure (mmHg)	Green flag: 62–88 Amber flags: 57–61 or 89–96 Red flags: below 57 or above 96	No changes – already aligned to national MEWS.
Temperature (degrees Celsius)	Green flag: 36.2–37.2 Amber flags: 35.7–36.1 or 37.3–37.4 Red flags: below 35.7 or above 37.4	No changes – already aligned to national MEWS.
Cardiac symptoms	Red flags: chest pain or shortness of breath or palpitations	Palpitations added.
Consciousness	Green flag: alert Red flags: CVPU (Confusion, Voice, Pain, Unresponsive)	Completely new parameters.
Vaginal blood loss less than 20 weeks	Amber flag: maternity sanitary pad not fully soaked within 30 min Red flag: maternity sanitary pad soaked within 30 min (50mls)	Amber flag added.
Symptoms less than 20 weeks	Amber flags: abdominal pain Red flags: shoulder tip pain or one sided abdominal pain or suspected/confirmed ectopic	Element changed to symptoms less than 20 weeks rather than pain. Amber and Red flags added.
Vaginal blood loss more than 20 weeks	Green flags: sticky, pink mucous plug (show) more than 37 weeks pregnant or clear fluid over 36 + 6 weeks Amber flags: sticky, pink, mucous plug (show) less than 37 weeks pregnant Red flag: any fresh red vaginal bleeding or blood stained amniotic fluid or meconium-stained waters (green) or offensive smelling waters or waters broken under 37 weeks	Element changed to PV loss rather just in relation to bleeding. Further red flags added in relation to amniotic fluid as well as blood loss. De-escalation of mucous show and term spontaneous rupture of membranes.
Abdominal pain	Green flag: none Red flags: constant abdominal pain (any gestation) or any pain or contractions 20–37 weeks or uterine scar pain (any gestation)	Element changed to abdominal pain. Red flag added for uterine scar pain.
Neurological	Green flag: asymptomatic Red flags: active seizure or history of recent seizure	Gestation removed from element to enable escalation of any seizure at any point during pregnancy. Recent seizure added.
Pre-eclampsia / eclampsia (more than 20 weeks gestation)	Green flag: nil Amber flag: nausea & vomiting or malaise Red flags: severe pain just below ribs or severe headache or problems with vision or sudden oedema to feet/hands/face	Entirely new element.
Appearance	Green flag: looks well Red flag: looks unwell	Entirely new element.
Additional concerns (no flag system)	Safeguarding concerns – follow local protocols	Entirely new part of the tool.

## Discussion

Prehospital maternity care is an underexplored area in research and often underrepresented in strategic maternity and neonatal programmes of work. Before this study, ambulance services did not have a tool to assess perinatal patients that have called the ambulance service with urgent or emergency obstetric or non-obstetric presentations [15]. Whilst tools have been developed to support prehospital assessment of other patient groups [4] or other settings for this patient group [7], our study shows these create a dual risk of misinformed reassurance and/or delayed escalation so are unsuitable. Therefore, in the absence of a suitable tool, consensus was required.

This study has shown that the modified Delphi approach is a helpful way to address patient safety concerns with minimal preexisting research and limited resources. The iterative process of developing a tool for a regional ambulance service and then scaling this up for national application has resulted in the creation of a tool which does not exist in literature or any other prehospital settings [15].

It must be acknowledged that the prehospital maternity decision tool is one element of supporting ambulance clinicians to provide perinatal patients safe care. This must be supplemented by appropriate and sufficient training [16], and an appetite to iteratively review and improve maternal care.

There is wide variance in funding for strategic posts relating to prehospital maternity care within UK ambulance services; such inconsistencies in quality improvement resourcing can contribute to health inequities [17, 18]. The development of this tool through the repeated modified Delphi approach highlighted the feasibility of working across systems to address inconsistences in care provision by ambulance services, and the benefits of triangulating learning across services [19]. Despite Coroners highlighting the need for consultant midwives in every ambulance service [20], this is still not the case in the UK. However, this work has demonstrated how collaboration, particularly where these specialist roles do exist, can lead to change nationally, supporting those services who are yet to adopt these roles.

Furthermore, the tool must also be used within a culture which supports escalation of care from prehospital to hospital setting. Referrals to alternative care pathways are encouraged in ambulance services to reduce inappropriate admission to hospital [21]. This paper shows that ambulance clinicians attending perinatal patients must be aware that they may be biased towards non-conveying patients who do not appear seriously unwell, which may lead to inappropriate triage of perinatal patients who may only have subtle signs of acute illness [3, 7]. This paper also shows that ambulance clinicians must not be measured according to non-conveyance data used for other patient groups.

It is also crucial that ambulance clinicians are empowered by systems, processes and partnership working to convey perinatal patients with confidence. The national Maternal Care Bundle [22] recommends the prehospital maternity decision tool must be integrated into electronic patient record systems so that ambulance clinicians are prompted to use the tool with every perinatal patient. The Northwest Ambulance Service has already implemented this and several UK ambulance trusts are currently undertaking this digitisation work. A national clinical audit of the tool to understand whether it is being consistently used has been proposed.

The Maternal Care Bundle [22] also aims to enhance the interface with maternity units to ensure prehospital teams can provide a pre-alert and receive an appropriate reception on their arrival. This study has shown the importance of using the tool to identify red flags, informing the maternity unit of the case via a pre-alert and promptly conveying for further assessment rather than calling a maternity unit to discuss patients prior to conveying, which could cause an altered level of concern and/or delays.

Finally, the tool must be used alongside clinical judgement. The most vulnerable perinatal patients accessing the ambulance service might not be aware of, or disclose pregnancy [3] so ambulance clinicians must use respectful professional curiosity whenever attending patients of childbearing age to understand whether it is possible they are perinatal and apply the prehospital maternity decision tool instead of NEWS2.

### Limitations

This is the first published study describing the development and implementation of a prehospital maternity decision tool on a national scale. However, the initial modified Delphi approach and data review was conducted in a single urban ambulance service, which has the potential to impact transferability due to the internal nuances of one organisation. The later modified Delphi involving representatives from other organisations across the UK minimises this risk.

In addition, due to the use of previously collected anonymised data, some cases had to be excluded from elements of the analysis because of missing data. These are clearly reported in the findings and reflect the completeness of clinical records in practice.

Due to the explorative nature of the study, a priori criterion for consensus was not defined. For the initial Delphi approach, focus group materials were not piloted in advance. However, the review served as the pilot for the second modified Delphi approach. Similarly, strategies to deal with non-consensus were not agreed in advance. However, at the outset attendees were asked to contribute according to their scope of practice and informed that any unresolved challenges could be fed back to the chair for consideration.

The modified Delphi approach was led by a clinician with experience of both hospital and prehospital maternity care to reduce any potential conflict of interest. As the modified Delphi approach was undertaken as focus group discussions, consensus evolved naturally. Therefore, it is not possible to report on results for each round separately, as would traditionally be seen with a Delphi approach.

### Future research

Future research is required, focused on validating the tool and evaluating its sensitivity and specificity. In addition, further investigation is needed to identify the factors associated with increased risk among women attended by the ambulance service. This would allow the system to explore the potential of refining the tool further to better account for the intersectionality of risk in deteriorating perinatal women. Finally, the tool was created for ambulance clinicians but further research is required to understand whether the tool would be helpful for other clinicians assessing perinatal patients outside of the hospital setting such as enhanced care teams, general practitioners and community midwives.

## Conclusion

This paper describes the iterative development of a bespoke prehospital maternity decision tool which is designed to provide ‘confidence in conveyance’ of perinatal patients across UK ambulance services. This paper demonstrates the benefits of cross-system working and wide stakeholder engagement in underrepresented areas of healthcare to address patient safety issues. The tool remains unvalidated and further research is required to understand the impact of the tool on decision making and outcomes.

## Supplementary Information

Below is the link to the electronic supplementary material.

Supplementary Material 1

## Data Availability

Data presented is held within the London Ambulance Service Clinical Audit and Research Unit. The dataset is available from the corresponding author on reasonable request.

## Abbreviations

MEWS: Maternity Early Warning Score
NEWS2: National Early Warning Score 2
SEIPS: Systems Engineering Initiative for Patient Safety
UK: United Kingdom
NHS: National Health Service
CREDES: Conducting and REporting of DElphi Studies missing refer ms and update
