# Supplementary material for: Prehospital assessment of perinatal patients by ambulance clinicians: development, implementation, review and national application
Source: Int J Emerg Med. 2026 Apr 16;19:107. doi: 10.1186/s12245-026-01158-5 (PMC13085477; doi:10.1186/s12245-026-01158-5)
Supplement: Supplementary file 1 — Supplementary Material 1 [file 12245_2026_1158_MOESM1_ESM.pdf]

- This tool should always be used when attending patients who are **pregnant** (or suspected pregnant) **regardless of gestation** or up to **4 weeks post birth/pregnancy loss/termination**.
- Use to aid immediate clinical decision making on scene.
- NEWS2 should not be used in this group of patients.
- This tool should be used alongside clinical judgement and other relevant JRCALC guidelines and local guidance/pathways.
- If there is a high concern (red flag), do not ask for remote advice from the maternity service, act on the concern(s) using the table below.
- Clinical parameters are relevant during labour and should not be dismissed due to pain. If birth is imminent, use the Imminent Birth guideline. Patient observations should be recorded during and after birth, any concerns highlighted by the tool should be acted on as soon as possible following birth.

	**High concern (red flag)**	**Medium concern (amber flag)**	**Low concern (green flag)**	**Medium concern (amber flag)**	**High concern (red flag)**
**Respiratory Rate (/min)**	Below 7	7-8	9-21	22-24	Above 24
**SpO_2 (%)**	Below 93%<br>Any oxygen requirement	93-94%	95-100%	N/A	N/A
**Pulse Rate (bpm) during pregnancy and up to 48 hours after birth**	Below 63	63-70	71-112	113- 121	Above 121
**Pulse Rate from 48 hours AFTER birth (bpm)**	Below 51	51-57	58-98	99-107	Above 107
**Systolic Blood Pressure (mmHg)**	Below 94	94-100	101-135	136-144	Above 144
**Diastolic Blood Pressure (mmHg)**	Below 57	57-61	62-88	89-96	Above 96
**Temperature (°C)**	Less than 35.7	35.7 - 36.1	36.2-37.2	37.3 - 37.4	Above 37.4

	**Low concern (green flag)**	**Medium concern (amber flag)**	**High concern (red flag)**
**Consciousness**	Alert	N/A	CVPU (Confusion, Voice, Pain, Unresponsive)
**Cardiac symptoms**	Nil	N/A	Chest Pain<br>Shortness of Breath<br>Palpitations
**Abdominal pain**	Nil	N/A	Any pain or contractions 20-37* weeks<br>Constant abdominal pain (any gestation)<br>Uterine scar pain (any gestation)
**Symptoms prior to 20 weeks pregnant**	Nil	Abdominal pain	Shoulder tip pain<br>One sided abdominal pain<br>Suspected/confirmed ectopic pregnancy
**PV blood loss LESS THAN 20 weeks pregnant**	Nil	Maternity sanitary pad not fully soaked within 30 minutes	Maternity sanitary pad fully soaked (50mls) within 30 minutes
**PV loss MORE THAN 20 weeks pregnant**	Sticky, pink, mucous plug (show) more than 37 weeks pregnant<br>Clear fluid over 36+6 weeks	Sticky, pink, mucous plug (show) less than 37 weeks pregnant	Any fresh red bleeding<br>Blood stained amniotic fluid<br>Meconium stained waters (green)<br>Offensive smelling waters<br>Waters broken under 37 weeks
**Postnatal PV blood loss (lochia)**	Maternity sanitary pad not fully soaked within 30 minutes	N/A	Maternity sanitary pad fully soaked (50mls) within 30 minutes<br>Offensive smelling lochia
**Pre-eclampsia/Eclampsia symptoms (MORE THAN 20 weeks gestation)**	Nil	Nausea & vomiting<br>Malaise	Severe pain just below ribs<br>Severe headache<br>Problems with vision<br>Sudden oedema to feet/hands/face
**Neurological**	Asymptomatic	N/A	Active seizure<br>History of recent seizure
**Appearance**	Looks well	N/A	Looks unwell

*Labour pain 37 weeks and above - refer to Imminent Birth guideline

Additional concerns:
Patient/family/clinician concerns should always be considered as part of the clinical assessment
Language barriers – use interpreting services and convey to hospital for further assessment
If recommended transfer is declined – escalate according to local protocols
Mental capacity concerns – follow local protocols
Safeguarding concerns – follow local protocols

	**All green flags**	**1 amber flag**	**Any red flag or 2 amber flags**
**Action required**	Follow local guidance.	Transport to hospital via ambulance.	Requires time critical assessment in hospital. Minimise time on scene.
**Emergency pre-alert**	No	Consider	Follow local guidance.
**Destination**	Follow local guidance.	Follow local guidance.	Follow local guidance.
**Ongoing observations**	If conveying continue regular observation	Observations every 15 minutes	Observations every 5 minutes
